# Synergistic Anti-Tumor Effects of Combination of Photodynamic Therapy and Arsenic Compound in Cervical Cancer Cells: In Vivo and In Vitro Studies

**DOI:** 10.1371/journal.pone.0038583

**Published:** 2012-06-08

**Authors:** Yong-Wan Kim, Su Mi Bae, Gantumur Battogtokh, Hyo Joo Bang, Woong Shick Ahn

**Affiliations:** 1 Catholic Research Institutes of Medical Science, The Catholic University of Korea, Seoul, Korea; 2 Department of Obstetrics and Gynecology, College of Medicine, The Catholic University of Korea, Seoul, Korea; Enzo Life Sciences, Inc., United States of America

## Abstract

The effects of As_4_O_6_ as adjuvant on photodynamic therapy (PDT) were studied. As_4_O_6_ is considered to have anticancer activity via several biological actions, such as free radical production and inhibition of VEGF expression. PDT or As_4_O_6_ significantly inhibited TC-1 cell proliferation in a dose-dependent manner (*P*<0.05) by MTT assay. The anti-proliferative effect of the combination treatment was significantly higher than in TC-1 cells treated with either photodynamic therapy or As_4_O_6_ alone (62.4 and 52.5% decrease compared to vehicle-only treated TC-1 cells, respectively, *P*<0.05). In addition, cell proliferation in combination of photodynamic therapy and As_4_O_6_ treatment significantly decreased by 77.4% (*P*<0.05). Cell survival pathway (*Naip1, Tert* and *Aip1*) and p53-dependent pathway (*Bax, p21^Cip1^, Fas, Gadd45, IGFBP-3* and *Mdm-2*) were markedly increased by combination treatment of photodynamic therapy and As_4_O_6_. In addition, the immune response in the NEAT pathway (*Ly-12, CD178* and *IL-2*) was also modulated after combination treatment, suggesting improved antitumor effects by controlling unwanted growth-stimulatory pathways. The combination effect apparently reflected concordance with in vitro data, in restricting tumor growth *in vivo* and in relation to some common signaling pathways to those observed *in vitro*. These findings suggest the benefit of combinatory treatment with photodynamic therapy and As_4_O_6_ for inhibition of cervical cancer cell growth.

## Introduction

Photodynamic therapy (PDT) involves the combination of non-toxic dyes known as photosensitizers (PSs) and visible light of the correct wavelength to be absorbed by the PSs. In the presence of oxygen, this leads to generation of reactive oxygen species (ROS) that can damage cellular constituents, leading to cell death [1], [2]. The discovery of programmed cell death, or apoptosis, has revolutionized the field of cytotoxic therapy in general, and PDT in particular [3]–[5]. However, a complete eradication of tumor cells by PDT alone has not been guaranteed [6]. Further study of controlling unwanted growth-stimulatory pathways after PDT is desirable to minimize the risk of harmful adverse effects.

Arsenical compounds have been demonstrated to possess life-preserving qualities in cancer treatment [7]–[10]. Promising results were reported, showing that diarsenic oxide (As_2_O_3_) treatment could offer an alternative to chemotherapy for acute promyelocytic leukemia (APL). Cytopathological studies also showed induction of apoptosis in APL cells. Recent reports suggested that arsenical compounds inhibit the proliferation of human umbilical vein endothelial cells (HUVEC) via G1 and G2/M phase arrest of the cell cycle [8]. In addition, the inhibitory effects on bFGF- or VEGF-stimulated cell proliferation suggest the antiangiogenic potential of arsenical compounds [11]. Furthermore, tetra-arsenic oxide (As_4_O_6_) was reported to have antiangiogenic effects on the NGF-induced formation of new vessels in the rat cornea, compared to control group and As_2_O_3_ treated group [9]. It has been therefore suggested that As_4_O_6_ might be a new arsenic compound, as it induced apoptosis in cancer cells at much lower concentration than As_2_O_3_ [10]. Arsenic compounds can increase reactive oxygen species (ROS) in cells and thereby increase apoptosis inducing factor (AIF) secretion through activation of PARP-1, and finally induce cell apoptosis. The release of cytochrome c and apoptosis inducing factor (AIF) is finely tuned by Bcl-2 family proteins in either of the following two ways: according to one possible mechanism, Bax present in the cytoplasm is translocated to the mitochondrial membrane, where it undergoes conformational changes assisted by Bid. The binding of Bax to the outer membrane (OM) causes its *in situ* multimerization, whereby PTP is gated and cytochrome c is released. Bcl-2 and Bcl-xL inhibit conformational changes in Bax and therefore inhibit release of cytochrome c and apoptosis. High level of ROS production is the main cause of apoptosis by arsenic compound. As free radical, ROS can react with most biological macromolecules, and therefore not only can induce oxidative damage on DNA, but also change structure and function of protein [12]–[14].

On the other hand, ROS can modulate genetic expression by acting as a second messenger. Several studies have suggested that oxidative damage might be involved in initiating events in cancer, and could help induce the initiation of apoptosis after an increase in cell proliferation [15], [16]. Arsenic compound could also reportedly regulate the immune response to involve anti-cancer function, through decrease of VEGF expression [12]–[14].

In this study, we firstly showed the enhanced anti-tumor effect of PDT using Radachlorin with As_4_O_6_ in mice bearing tumors caused by human papillomavirus (HPV) 16 E6/E7 oncogene expressed TC-1 tumor cells. The present study showed that the combination therapy of PDT plus As_4_O_6_ was much more effective on the suppression of tumor growth, compared with PDT or As_4_O_6_ alone.

## Results

### In vitro cell growth inhibitory effect of As_4_O_6_ plus Radachlorin/PDT on TC-1 cells

**Figure 1:**
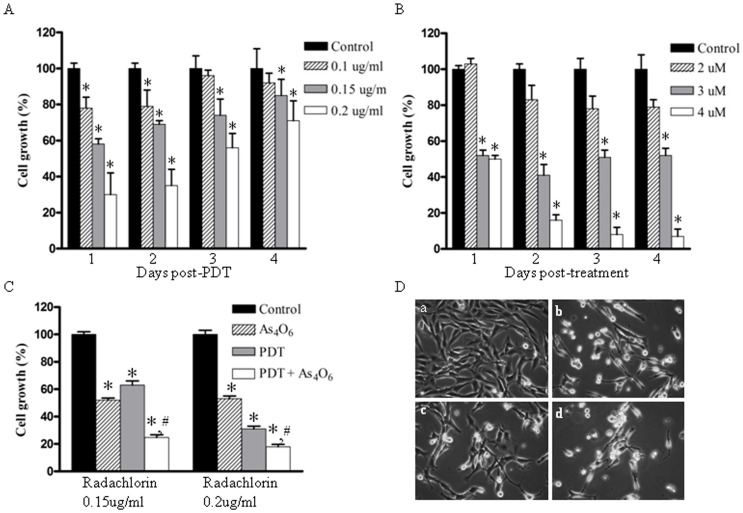
Cell growth inhibition effects of photodynamic therapy and As_4_O_6_. (A) TC-1 cells (3×10^3^) were cultured in 12-well plates in triplicate overnight and treated with different concentrations of Radachlorin and PDT (6.25 J/cm^2^) as described in Materials and Methods. After PDT, the cells were cultured for a predetermined time. (B) Inhibition effects of cell growth of As_4_O_6_ on TC-1 cells. TC-1 cells were cultured and treated as described in Materials and Methods. After As_4_O_6_ treatment, the cells were cultured for a predetermined time. Cell viability was determined based on the MTT assay. (C) *In vitro* cell growth inhibitory effects of As_4_O_6_ plus Radachlorin/PDT on TC-1 cells. TC-1 cells were cultured with 3 uM of As_4_O_6_ and different doses of Radachlorin/PDT for 1 day, as described above. Cell viability was determined based on the MTT assay. Each bar represents a mean [± SD (vertical line)] of three replicates per dose (*n* = 3). * and #: significantly different (*P*<0.05) from the control and the PDT by the student’s t-test. (D) Morphological changes of TC-1 cells. TC-1 cells were treated with 0.15 ug/ml Radachlorin/PDT or/and 3 uM of As_4_O_6_ for 1 day. Cells were then viewed under microscope. Pictures were taken with phase contrast microscopy at X300. (a) non-treated; (b) 0.15 ug/ml Radachlorin/PDT alone; (c) 3 uM As_4_O_6_ alone; (d) 3 uM As_4_O_6_ plus 0.15 ug/ml Radachlorin/PDT.

**Figure 2:**
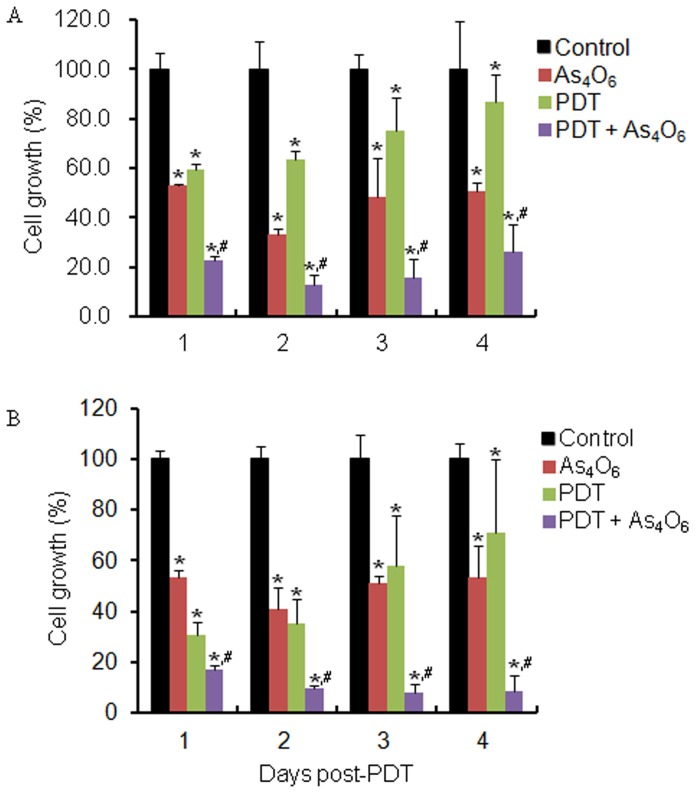
In vitro cell growth inhibitory effects of As_4_O_6_ plus Radachlorin/PDT on TC-1 cells. TC-1 cells were cultured with 3 uM of As_4_O_6_ and (A) 0.15 ug/ml and (B) 0.2 ug/ml of Radachlorin/PDT, respectively. Cell viability was determined based on the MTT assay. Each bar represents a mean [± SD (vertical line)] of three replicates (n = 3). * and #: significantly different (P<0.05) from the control and the PDT by the student's t-test.

To see the growth inhibition effect of PDT on TC-1 cell, the light of 6.25J/cm^2^ was exposed at 12 hr after Radachlorin treatment on the cells, and then the cell growth was measured for a predetermined time. Viability of cells treated with various doses of Radachlorin followed by light irradiation was reduced in a dose dependent manner compared to control, respectively (Figure 1A). To see the growth inhibition effect of As_4_O_6_ on TC-1 cell, the cell growth was measured for a predetermined time after As_4_O_6_ treatment. Viability of cells treated with various doses of As_4_O_6_ was reduced in a dose dependent manner compared to control, respectively (Figure 1B). Using these data, the viability of cells was determined after treating the cultured TC-1 cells with 3 uM of As_4_O_6_ and different doses of Radachlorin/PDT per day. The combination treatment showed synergistic effect, decreasing viability in a dose dependent manner as compared to control, as shown in Figure 1C. Cell viability was found to be 62.4% for PDT alone and 52.5% after As_4_O_6_ alone treatment at a low dose. In contrast, after PDT plus As_4_O_6_ treatment, the percentage of cell growth was found less than 23%. We also observed the combined effect vs. single doses over time to elucidate whether the combinatory approach can result in longer-lived restriction of cell proliferation compared to individual therapies (Figure 2). Cell viability was found to be less than 10% for 0.2 ug/ml of Radachlorin/PDT plus As_4_O_6_ treatment for three days, as compared to individual treatment. For the evaluation of synergism between Radachlorin/PDT and As_4_O_6_ treatment, we used a combination index that calculated by Chou and Talalay's method (Table 1). Among the several combinations of treatment, 0.2 ug/ml Radachlorin/PDT plus 3 uM of As_4_O_6_ on day 3 and 4 led to the highest cell death rate and it showed synergism. A few more cell lines such as HaCaT, HeLa, and SiHa cells were included for evaluating the inhibition of cell growth (Supplement Figure S1). While the results of HaCaT and SiHa were consistent with earlier estimates of MTT assay, HeLa showed Radachlorine/PDT-resistant trend compared to the other cells. The cell viability of the two cell line was found to be less than 25% for 0.15 ug/ml of Radachlorin/PDT plus As_4_O_6_ treatment for four days, as compared to individual treatment. We characterized cell death by staining the TC-1 cells treated with 0.15 ug/ml Radachlorin/PDT or/and 3 uM of As_4_O_6_ for 1 day. As expected, the effect of the combination treatment was larger than each single treatment. In the absence of Radachlorin/PDT or As_4_O_6_, the cells achieved a completely confluent, dense monolayer after 48 h of culture (Figure 1D). The cells remained attached to the tissue culture substrate and they adopted an elongated morphology. In contrast, the majority of the cells treated with Radachlorin/PDT plus As_4_O_6_ was detached from the plate and was rounded; characteristic of cells undergoing death by apoptosis. The cells treated with Radachlorin/PDT or As_4_O_6_, however, adopted morphologies that were intermediate in nature. We counted different apoptotic cell populations induced by 0.15 ug/ml Radachlorin/PDT or/and 3 uM of As_4_O_6_ for 1 day. As shown in Supplement Figure S2, the cell death significantly increased after Radachlorin/PDT plus As_4_O_6_ treatment. Early apoptotic population was 9.9% at Radachlorin/PDT plus As_4_O_6_ treatment. In contrast, early apoptotic cell populations were 4.3% and 4.1% at Radachlorin/PDT and As_4_O_6_ treatment, respectively. This shows that the combination treatment induced more early apoptotic cells compared to individual therapies.

**Table 1:** Combination index (CI) values for TC-1 cells treated with Radachlorin/PDT and As_4_O_6_.

		Day 1		Day 2		Day 3		Day 4	
As_4_O_6_	PDT	f*(%)	CI**	f(%)	CI	f(%)	CI	f(%)	CI
3uM	0.2ug/ml	17.3	1.61	9.7	1.16	8.2	0.98	8.7	0.67
3uM	0.15ug/ml	22.6	1.52	12.7	1.13	15.7	1.02	26.4	1.19

The combination index (CI) values were calculated using the Chou and Talalay mathematical model for drug interaction on Calcusyn software. A CI was equal to 1 denotes additivity; antagonism if the CI>1; CI values between 1 and 0.7 indicate slight synergism; 0.7 to 0.3, synergism; <0.3, strong synergism. *Viability.

### Gene expression profile of TC-1 cells treated with As_4_O_6_ plus Radachlorin/PDT

**Figure 3:**
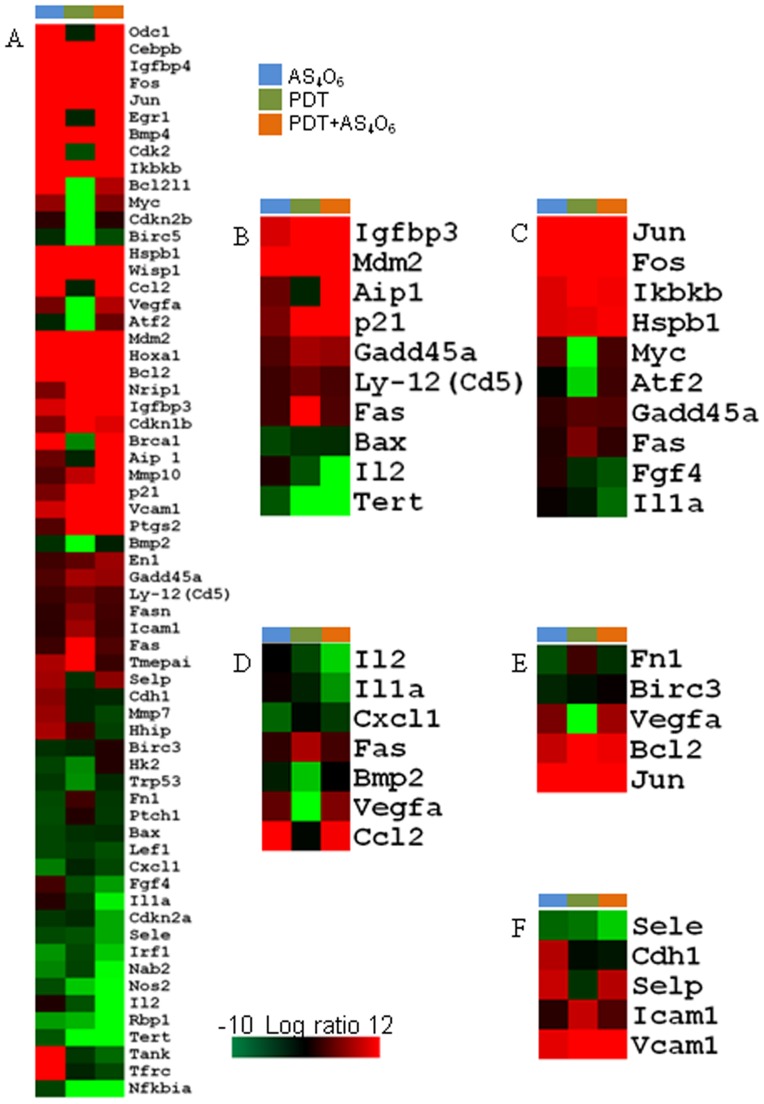
Hierarchical cluster analysis. (A) All the data were median centred and clustered using a hierarchical clustering. A cluster image representing 63 of the genes is shown in matrix format, where rows represent individual genes and columns represent each assay. Each cell in the matrix represents the expression level of a gene in an individual assay. Red and green cells reflect high and low expression levels, respectively. (B) Ten genes (p53 and NEAT pathways) showing statistically significant differences in three groups are shown as a cluster image. (C) MAPK pathway as a cluster image. (D) Cytokine-cytokine receptor interaction pathway. (E) Focal adhesion pathway. (F) Cell adhesion pathway.

**Figure 4:**
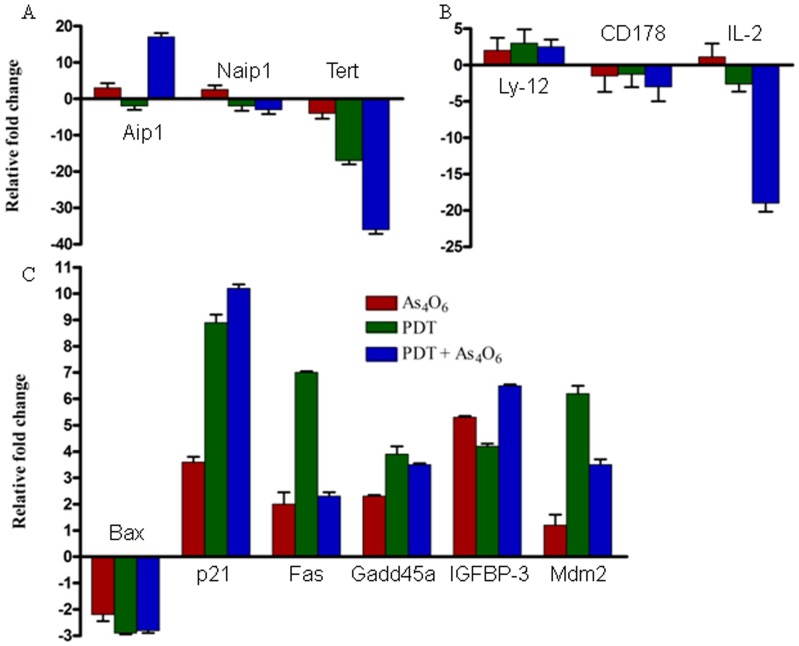
*In vitro* gene expression profiles using RT^2^Profiler^TM^ PCR array of TC-1 cells treated with 0.15 ug/ml Radachlorin/PDT or/and 3 uM of As_4_O_6_ for 1 day. The results are presented as transcript levels relative to the level in untreated control cells by using the CT method, with average mRNA level of five internal control genes, including b-actin, used as the normalization control. (A) Cell survival pathway. (B) NEAT pathway. (C) p53 pathway.

We used RT^2^ Profiler PCR Array System to understand the cellular process changes through which TC-1 cells could be influenced by the combination treatment with As_4_O_6_ plus Radachlorin/PDT. We found 63 genes which were at least two fold up- or downregulated (each gene is detailed in Table 2) and used hierarchical clustering to show differentially expressed genes (Figure 3A). To detect the differences in the functional profiles, we placed differentially expressed genes in the context of present interactome knowledge, using the Ingenuity Pathways Analysis tools (*P* for all <0.05), showing that the enhanced cell growth inhibition and antitumor effects were significantly related with gene expression levels of genes related with cell death, i.e. p53 and NFAT pathway (Figure 3B). Each gene in the pathway was tested by quantitative PCR (Figure 4) and the results of the PCR array and qRT-PCR were similar. These included genes coding for Tert, Aip1, Bax, p21, Fas, Gadd45, IGFBP-3, Mdm-2, Ly-12, and IL-2, as compared to slightly expressed genes such as Naip1 and CD178. Significantly up-regulated molecular functions for the combination treatment group was p53 pathway. The down-regulated molecular function was NEAT pathway. MAPK, cytokine-cytokine receptor interaction, focal adhesion, cell adhesion pathways were also studied in the gene sets, but not strictly correlated with the enhanced cell growth inhibition (*P* for all >0.05) (Figure 3C–F).

**Table 2:** Gene expression profiles of TC-1 cells treated with 0.15 ug/ml Radachorin/PDT and/or 3 uM of As_4_O_6_ for 1 day.

Symbol	As_4_O_6_	PDT	PDT + As_4_O_6_	Symbol	As_4_O_6_	PDT	PDT + As_4_O_6_
Odc1	13064.2	−1.1	9131.7	Icam1	1.4	5.0	1.9
Cebpb	6309.6	384.5	4319.6	Tmepai	5.4	8.6	1.8
Igfbp4	4683.4	7315.1	2413.1	Cdkn2b	1.4	−262.0	1.2
Egr1	1317.3	−1.1	1495.8	Birc3	−1.5	−1.2	1.1
Fos	1513.1	5281.3	1043.1	Hk2	−2.1	−4.3	1.1
Jun	977.8	4927.6	1035.9	Bmp2	−1.5	−14.2	−1.0
Bmp4	964.3	1000.6	414.9	Cdh1	4.3	−1.1	−1.2
Cdk2	254.8	−2.4	371.4	Trp53	−1.7	−4.5	−1.3
Hspb1	67.8	81.4	118.3	Bax	−2.2	−1.5	−1.4
Ikbkb	68.8	279.5	106.6	Fn1	−2.3	2.0	−1.7
Wisp1	68.8	150.8	56.0	Ptch1	−2.4	1.1	−1.8
Mdm2	12.1	71.8	46.1	Hhip	5.3	1.7	−2.0
Hoxa1	17.4	45.2	32.6	Tfrc	725.8	−1.1	−2.2
Brca1	8.0	−4.2	31.3	Mmp7	4.8	−1.1	−2.2
Ccl2	108.6	−1.1	27.8	Cxcl1	−3.9	−1.1	−2.2
Aip 1	3.3	−1.1	17.0	Birc5	−1.4	−229.7	−2.4
Bcl2	8.5	32.8	13.3	Lef1	−2.2	−1.7	−2.5
p21	3.8	12.0	13.1	Tank	40.3	−1.7	−3.3
Mmp10	2.5	6.0	12.7	Fgf4	2.1	−2.4	−4.9
Vcam1	6.5	12.4	10.6	Cdkn2a	−1.7	−1.3	−5.3
Nrip1	3.9	35.9	10.3	Sele	−2.3	−2.6	−5.3
Ptgs2	2.6	9.8	9.5	Irf1	−4.7	−2.2	−6.3
Igfbp3	6.8	44.8	8.0	Il1a	1.2	−1.6	−7.5
Cdkn1b	4.0	43.0	7.0	Nab2	−4.2	−2.1	−7.9
Vegfa	3.7	−76.3	5.6	Nos2	−2.4	−6.3	−12.7
Bcl2l1	10.7	−170.5	5.6	Il2	1.0	−2.6	−17.2
En1	2.0	3.0	4.9	Rbp1	−4.9	−5.7	−19.3
Gadd45a	2.5	5.2	4.7	Tert	−2.7	−17.2	−34.6
Selp	5.1	−1.5	4.4	Nfkbia	−2.1	−1055.2	−2125.1
Myc	4.6	−174.0	3.9				
Atf2	−1.1	−55.5	3.3				
Fas	1.9	10.1	2.5				
Ly-12(Cd5)	1.9	3.3	2.3				
Fasn	1.5	4.2	2.1				

### As_4_O_6_ plus Radachlorin/PDT suppresses tumor growth in TC-1 animal model

**Figure 5:**
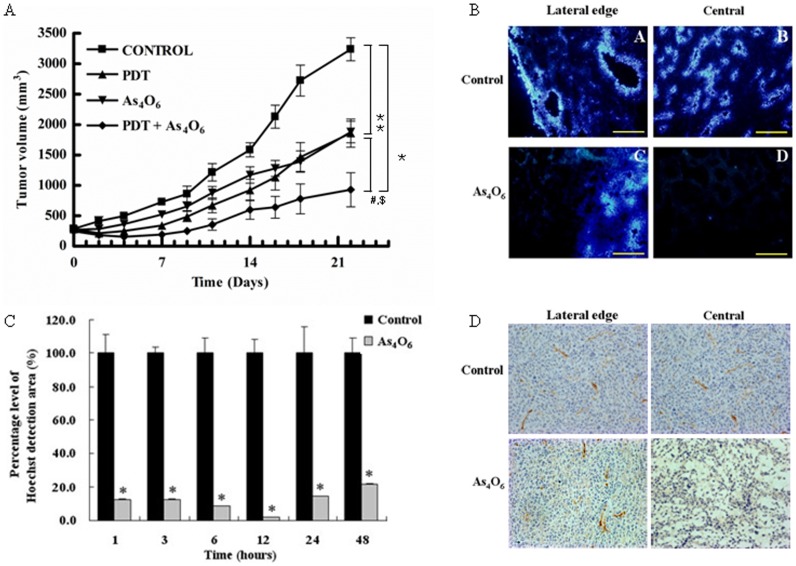
*In vivo* effect of the combination of As_4_O_6_ and Radachlorin/PDT on tumor growth inhibition in TC-1 cell-challenged C57BL/6 mice. (A) Tumor growth curves for TC-1 of mice treated with Radachlorin/PDT and/or As_4_O_6_ (n = 7 for each group). Tumor bearing mice were given intravenously injected Radachlorin (10 mg/kg b.w.) and/or peritoneal injections of 7.5 mg/kg of As_4_O_6_. Three hours later, cells were exposed to laser, tumor size was monitored thereafter, as described in Materials and Methods, and its mean ellipsoid volume was plotted over time. Significant inhibition of tumor growth was detected by ANOVA. *: *P*<0.01, compared with the control group, #: *P*<0.01, compared with the Radachlorin/PDT alone group and $: *P*<0.01, compared with the As_4_O_6_ alone group. (B) Perfusion and morphological changes in TC-1 tumors in C57BL/6 mice treated with As_4_O_6_ (10 mg/kg b.w., i.p.). Tumor tissues were harvested after 48 h after As_4_O_6_ treatment. Tissue were viewed at a wavelength of 365 nm and photographed at x200 magnification. (C) Percent area of perfusion area of TC-1 tumors in C57BL/6 mice treated with As_4_O_6_ compared with non treated tumors. The tumor tissues were harvested at indicated times after As_4_O_6_ treatment. Sequential changes of perfused areas at 1, 3, 6, 12, 24, and 48 hr after As_4_O_6_ treatment. *P<0.05, compared to untreated controls. (D) CD31-immunostained tumor sections (original magnification, x400) and As_4_O_6_-treated sections are displayed. Twenty-four hours after As_4_O_6_ treatment, TC-1 tumors showed extensive loss of CD31 staining indicative of significant As4O6-induced vascular damage.

To determine the synergistic antitumor effects of As_4_O_6_ plus Radachlorin/PDT *in vivo*, C57BL/6 mice were challenged s.c. with TC-1 cells and then treated with As_4_O_6_ or/and Radachlorin/PDT. Treatment was initiated on day 0 when tumors were ∼230 mm^3^ in size. As shown in Figure 5A, As_4_O_6_ or Radachlorin/PDT treated animal groups showed significant suppression of tumor growth, compared to the untreated control group. However, animals treated with combination of As_4_O_6_ plus Radachlorin/PDT showed the most significant tumor suppression, compared to the other groups. This suggested that As_4_O_6_ may play an important role in inhibiting the growth by Radachlorin/PDT.

### Effect of arsenic treatment on regional blood perfusion in tumor tissue

Hoechst 33342 was used to visualize perfused vessels as described in material and methods. In control tissue, vascular endothelial cells were clearly stained by Hoechst 33342 at both their outer edge and central region (Figure 5B). Treated tissue was centrally destained and the periphery florescence signal was almost undetectable. This phenomenon continued to the 24 h point. However, fluorescence at the periphery of cells was clearly evident at 48 h post-treatment, while central staining still remained absent. In order to observe changes of vessel function in whole tumor, tumor section were scanned field by field. The sequential perfusion area changes after As_4_O_6_ treatment are shown in Figure 5C. At each time point, the percentage of every section to tumor field was comparable to control tissue at each time point. The perfusion area was reduced immediately after As_4_O_6_ treatment. At 6 and 12 hr, the perfusion area was the minimum, at 24 h the perfusion area was 14.4% compared with tissue from PBS treated control tissue. Tumor microvessel density dimensions were measured after the administration of As_4_O_6_ to mice bearing TC-1 tumor xenografts. As shown in Figure 5D, there was a significant difference in tumor vessel density as detected by CD31 staining between the As_4_O_6_-treated group and the time-matched PBS treated control group after 24 hr of As_4_O_6_ treatment. Mice treated with As_4_O_6_ had a reduction in tumor vessel dimensions compared with the control animals, with CD31 stained microvessels still being apparent at the outer edge of the tumor.

### In vivo gene expression levels of tumor tissues treated with As_4_O_6_ plus Radachlorin/PDT

**Figure 6:**
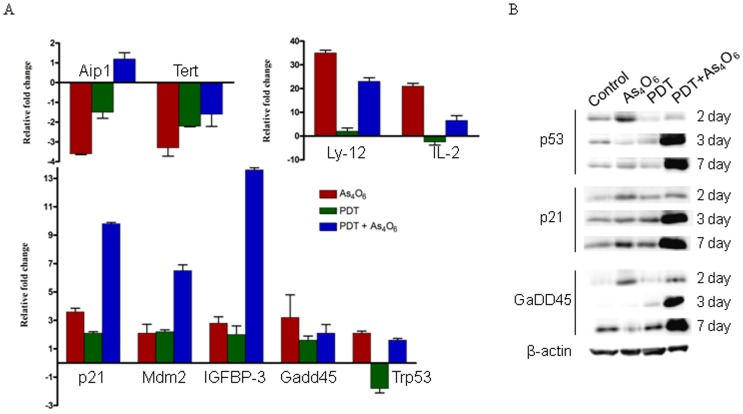
*In vivo* gene expression profiles in tumor tissues treated with 0.15 ug/ml Radachlorin/PDT or/and 3 uM of As_4_O_6_ for 1 day. (A) The expression of genes mRNA in tumor tissues was examined by Q-PCR. The results are presented as transcript levels relative to the level in untreated control cells by using the CT method, with b-actin mRNA levels used as the normalization control. (B) Western blot analysis of in vivo tumor tissues treated with 0.15 ug/ml Radachlorin/PDT or/and 3 uM of As_4_O_6_ for several days.

TC-1 cells were treated with As_4_O_6_ and Radachlorin/PDT for 1 day, as indicated in the “Materials and Methods” section. The expression of genes mRNA in tumor tissues was examined by qRT-PCR (Figure 6A). The results are presented as transcript levels relative to the level in untreated control cells by using the CT method, and b-actin mRNA levels were used as the normalization control. Using CD5 antigen in the combination therapy group, Ly-12 and IL-2 gene expression increased 26 and 17 fold, respectively, compared to the control group, showing that p53 was involved in regulating the NFAT pathway of p21, Gadd45, IGFBP-3, Mdm-2 and other genes. Gene expression was found increased in three consecutive experiments. To determine the roles of p53 signaling pathways in the synergistic antitumor effects of As_4_O_6_ plus Radachlorin/PDT *in vivo*, we further investigated the p53, p21, and Gadd45 protein levels in vivo (Figure 6B). As_4_O_6_ or Radachlorin/PDT treated animal groups showed no significant expression changes, compared to the untreated control group. In contrast, p53, p21, and Gadd45 protein were significantly increased in the combination treatment with As_4_O_6_ plus Radachlorin/PDT.

## Discussion

In this study, we comparatively analyzed results of single treatment of As_4_O_6_ or photodynamic therapy groups with those obtained by the co-treatment using E6/E7 expressing TC-1 cell line and C57BL/6 mouse model transplanted with this cell. We finally report the enhanced cell growth inhibition and antitumor effects and changes in gene expression levels of genes related with cell death, i.e. p53 and NFAT pathway, induced by this As_4_O_6_ and PDT co-treatment method.

These effects were produced by the photodynamic therapy depending on the concentration of the photosensitizer Radachlorin, and by the As_4_O_6_ treatment in a concentration dependent manner. In the PDT-untreated cells, the cell growth was consistently maintained until the end of the observation period. PDT-treated cells were regressed only for 1 day post-PDT, but the cell growth was re-increased after 2 days post-PDT. The lower the As_4_O_6_ level was, the slower the cell growth inhibiting effect was shown. Under 3 uM of As_4_O_6_, cell growth inhibition seemed to begin on the 1st day of treatment and was consistently maintained under 60∼80% level of cell growth during the first 4 days of treatment. In contrast, concentrations of As_4_O_6_ higher than 3 uM seemed to be cytotoxic. In the co-treatment group of As_4_O_6_ and PDT, more significant effects on cell growth inhibition and more morphological changes were found, compared to each single-treatment group. It has been known that antitumor agents generate reactive oxygen and reactive nitrogen species to induce apoptosis and necrosis of the cell [17]–[19]. As one of the antitumor agents, the antitumor mechanism of arsenic compound to generate reactive oxygen and nitrogen species in the colorectal cancer cell line has also been reported [20]. The supply of sufficient oxygen and the generation of reactive oxygen species (ROS) are known to be significant factors determining the effect of photodynamic therapy [4], [21]. Therefore, induction of ROS mediated by antitumor agents might have contributed to enhancing the efficiency of PDT.

Next, we comparatively analyzed the change of intracellular signal pathway of each single-/co-treatment group, using the RT^2^Profiler^TM^ PCR array method, and those results were confirmed by the real-time PCR examination using 13 different primers. Using the real-time PCR method, we analyzed the change in expression levels of 84 genes involved in 15 signaling pathways. We found significant changes in the expression levels of genes known to be involved in cell survival pathway (e.g., Naip1, Tert, Aip1, etc.), p53 pathway (e.g., Bax, p21, Fas, Gadd45, IGFBP-3, Mdm-2, etc.), and NFAT (nuclear factor of activate T-cell) pathway (e.g., Ly-12, CD178, IL-2, etc.) involved in modulation of the immune response. Especially, expression levels of Naip1, which is known as the apoptosis inhibitory gene, and Tert, the telomerase reverse transcriptase, were decreased in every cell survival pathway, whereas the expression level of the apoptosis inducing gene Aip1 was enhanced. Moreover, the PCR array results showed decreased levels of Tert gene expression of 2.7, 17.1, and 34.6 fold in As_4_O_6_ alone, PDT alone, and co-treatment groups, respectively, each compared to the control group. Tert gene is known to be related with activation of telomerase, and in case of cervical cancer, the c-myc gene seems to induce telomerase activation by Tert expression [22]. Therefore, we suggest that down-regulation of the Tert gene induced by this co-treatment of As_4_O_6_ and PDT might have been involved in cell death. The inhibition of Tert gene was also confirmed by the real-time PCR examination performed after RT^2^ Profiler^TM^ PCR array analysis, and this reduction of Tert gene expression level was consistently shown in the co-treatment group, both *in vitro* and *in vivo*.

We also observed changes in the expression levels of p21, Fas, Gadd45, IGFBP-3, and Mdm-2 genes, known to be involved in the p53 pathway, and the co-treatment group showed more significant increase compared to each single-treatment group. However, any significant change of Bax gene expression (known to be activated by p53) was not observed. Moreover, from the absence of significant changes of p53 level, those changes of p21, Fas, Gadd45, IGFBP-3, and Mdm-2 gene expression levels seem to be mediated by p53-independent pathways.

In addition, considering the expression levels of genes like Ly-12, CD178, and IL-2, known to be involved in the NFAT pathway, Ly-12 expression increased, whereas CD178 and IL-2 expressions rather decreased in the co-treatment group, compared to each single-treatment group. As the intracellular pathway of NFAT has been known to be associated with increased immune responses, we expected that it might be up-regulated by PDT; however, the gene expression levels in this pathway, including IL-2, decreased after PDT treatment and further decline was found in the co-treatment group. However, when we tried to confirm the results of RT^2^Profiler^TM^ PCR array using the real-time PCR method, we found opposite results compared to those of RT^2^Profiler^TM^ PCR array, i.e. IL-2 expression decreased in the PDT alone treatment group and increased in the co-treatment group, both showing similar results under *in vitro* and *in vivo* conditions. It was reported that PDT might stimulate tumor growth-promoting immune signals and could be improved by controlling unwanted growth-stimulatory pathways [23], [24]. Therefore, our results for IL-2 seem to show that PDT decreased the expression of this gene, whereas it was increased by the arsenic compound treatment, indicating that the combination therapy combine two advantages by inducing immune mediators and antitumor effects. In addition, this concurrent treatment increased the *in vitro* and *in vivo* expression levels of the CD5 antigen gene and Ly-12 up to 23 and 26 fold, respectively. However, expression levels of Ly-12 or IL-2 did not show significant changes in the PDT alone treatment group; therefore, the increased gene expression seemed to be induced by the arsenic compound. So far, however, no other studies have reported the relationship between the combined PDT-As_4_O_6_ treatment and the NFAT pathway, and more intensive studies should be carried out.

Similarly, our TC-1 cell line-transplanted mouse model showed significantly enhanced antitumor effect in the PDT and As_4_O_6_ co-treatment group, compared to each single-treatment group. To identify the mechanism of this effect, we performed real-time PCR experiment using 10 different primers and finally observed significant changes in the expression levels of genes known to be involved in the cell survival pathway, p53 pathway, and NFAT pathway, similar with those obtained from *in vitro* experiments. Our western blot data demonstrates that co-treatment increased expression of p53, p21, Gadd45 at 7 day compared to single-agent treatment and control. Activation of p53 protein plays a crucial role in the control of tumor cell response to chemotherapeutic agents and DNA-damaging agents. p21 and GaDD45, downstream effectors of p53, were activated in combination treatment producing apoptosis in TC-1 tumor cells. These results suggest that apoptosis may be mediated through the p53 signaling pathway via up-regulation of proapoptotic proteins.

During PDT, intracellular depletion of oxygen and collapsed blood vessels might induce intracellular hypoxia and this could up-regulate the level of VEGF expression to catalyze angiogenesis [25]. Many studies on PDT have been trying to increase the efficiency of this method by supporting the generation of intracellular ROS, or by performing this method in combination with several inhibitors of angiogenesis, such as COX-2, MMP, or VEGF [9], [26]–[31]. Arsenic compounds are currently used as therapeutic agents for acute leukemia and various solid tumors, and they also have been reported to generate ROS and inhibit the VEGF expression [17], [19]. In this study, the vascular disruption effect of As_4_O_6_ was determined by Hoechst33342 perfusion and CD31 immunohistochemistry staining. Blood perfusion area decreased rapidly after As_4_O_6_ treatment. From the 3 h time point, the central part of tumors disappeared with respect to perfusion activity and, finally, almost no signal could be detected in the whole tumor area, even the outer edge. Interestingly, this reduction of the perfusion area was recovered from the 24 h time point, and the florescence appeared from the outer edge clearly at 48 and 72 h. Even though the blood perfusion appeared to be recovered from the outer edge 72 h after treatment, no signal was detected from inside of the tumors. These data showed that much more intensive immunoreactive microvessels were observed in tumor tissues from mice treated with PBS, but little CD31 staining was present in the central region of tumor tissues from mice treated with As_4_O_6_. The results echo those of studies involving As_2_O_3_ and Conbretastatin A-4 [32], [33]. As_2_O_3_ decreased blood perfusion relative to controls up to 6 h after As_2_O_3_ treatment with some recovery at 24 h [32], and also CA-4-P, 24 h after treatment on P22 tumors vascular functions displayed partial recover [33]. Therefore, in this study, we used one of the arsenic compounds, i.e. As_4_O_6_, in combination with the PDT method to investigate the increasing antitumor effect and also its intracellular signaling pathways. These data showed that the effect of the combinatory therapy on VEGF and associated pathways were strengthened with the assessments of tumor-associated vasculature in the animal model and of angiogenesis markers in the tumor lesions. We finally found that changes in the expression levels were important, particularly of genes known to be involved in the cell survival pathway, p53 pathway, and NFAT pathway involved in modulation of the immune response. This co-treatment of As_4_O_6_ and PDT induced significantly enhanced antitumor effects, both *in vitro* and *in vivo*, compared to those of each treatment alone. This seems to indicate that the presence of As_4_O_6_ might increase the antitumor effect of PDT by inducing changes in the expression of genes involved in cell survival, p53, and NFAT pathways. In the future, this co-treatment method of PDT and As_4_O_6_ may lead to improved results of antitumor therapy.

## Materials and Methods

### Ethics Statement

All procedure of animal research were provided in accordance with the Laboratory Animals Welfare Act, the Guide for the Care and Use of Laboratory Animals and the Guidelines and Policies for Rodent experiment provided by the IACUC (Institutional Animal Care and Use Committee) in school of medicine, The Catholic University of Korea [permit no: CUMC-2008-0061-02].

### Cell cultures

TC-1 cells prepared by transformation of C57BL/6 primary mouse lung cells with HPV16 E6/E7 oncogene and activated H-RAS as have been kindly provided from the cell line bank at Johns Hopkins University (a kind gift from Dr. Wu, Johns Hopkins University, MD, USA). The cells were routinely propagated in monolayer cultures in RPMI-1640 medium, supplemented with 5% heat-inactivated fetal bovine serum, 0.22% sodium bicarbonate, and penicillin/streptomycin. The cells were cultured in a 5% CO_2_ incubator at 37°C.

### Photosensitizer and laser

The PDT was carried out using a diode laser generator apparatus (Won-PDT D662, Won Technology, Daejon, Korea) equipped with a halogen lamp, a band-pass filter and a fiber optics bundle. The wavelength was set at 662± 2 nm. Under PDT treatment, duration of the light irradiation was calculated taking into account the effective dose of light energy in J/cm^2^. Radachlorin (RADA-PHARMA Co, Ltd., Moscow, Russia) was used as a photosensitizer.

### Arsenic compound

As_4_O_6_ was provided by Chonjisan Co. (Seoul, Korea). These chemicals were diluted in phosphate-buffered saline (PBS) and kept at 4°C.

### MTT assay

To assess cell viability by PDT plus As_4_O_6_, TC-1 cells (3×10^3^) were treated with 3 uM of As_4_O_6_ and/or 0.15 ug/ml, and 2.0 ug/ml of Radachlorin (RADA-Parma). After incubation for 12 hrs, the cells were washed with fresh medium and exposed to PDT (6.25 J/cm^2^). The cells were then further incubated at 37°C for 1, 2, 3, or 4 days, in a humidified incubator. After incubation with PDT plus As_4_O_6_ co-treatment, 100 ul of 3-(4,5-dimethylthiazol-2-yl)-2,5-diphenyltetrazolium bromide (MTT) solution (2 mg/ml) was added to each well and cultured for 4 h. One hundred microliters of dimethyl sulfoxide (DMSO) was added to each well for 10 min, and the absorbance was measured with an automated spectrophotometric microtiter plate reader (SpectraMax 340; Molecular Devices, Sunnyvale, CA), using a 570-nm filter. The morphological changes of the cells were determined by optical microscopy.

### Evaluation of in vitro combination effects by the Chou-Talalay method

The combined effects of Radachlorin/PDT and As4O6 on cell survival were analyzed using Chou-Talalay method, which applies the median-effect equation of Chou and the CI equation of Chou and Talalay [34]. TC-1 cells were plated in 96-well microplates as above were exposed in triplicate to each agent or both in combination using the constant ratio combination design for 96 hours, followed by the MTT assay for cell viability determination. Calculated CIs were used to ascertain the presence of strong synergism (CI<0.3), moderate synergism (0.3< CI<0.9), additive effect (CI = 1), antagonism (CI>1) and strong antagonism (CI>3.3) between Radachlorin/PDT and As4O6.

### FACS analysis

TC-1 cells were washed twice with PBS and then resuspended in 1X binding buffer (10 mM HEPES/NaOH, pH 7.4, 140 mM NaCl, 2.5 mM CaCl2). Then, 1×105 cells per tube were added with 5 μl of Annexin V-FITC and 10 μl of propidium iodide (BD, San Jose, CA), followed by incubation at 22°C for 15 min. Each tube was added with 100 μ of 1X binding buffer and then the cells were analyzed by a flow cytometer (BD). The samples were read using flow cytometer (BD). Cell debris and fixation artifacts were gated out using the CellQuest program.

### Real-time PCR microarray analysis with TC-1 cell

TC-1 cells were treated with 3 uM of As_4_O_6_ and/or 0.15 ug/ml of Radachlorin (RADA-Parma). After incubation for 12 hrs, the cells were washed with fresh medium and exposed to light. The following four groups were used in this study: control, 3 uM of As_4_O_6,_ 0.15 ug/ml of Radachlorin/irradiation, and 3 uM of As_4_O_6_ plus 0.15 ug/ml of Radachlorin/irradiation. For all groups, cells were harvested 24 h after irradiation. Total RNA was isolated from cells according to the manufacturer's recommendations using TRIzol and the Absolutely RNA kit from Stratagene. RNA (2 μg) was reverse transcribed in a total volume of 20 μl, using 200 U of Superscript II (Invitrogen) reverse transcriptase, 100 pmol oligo-dT, 0.5 mM dNTP, and 40 U RNasin (Promega). The resultant cDNA was diluted 1∶100 with nuclease-free water. Five microliters of diluted cDNA was used in subsequent PCR reactions. Samples for each group were analyzed according to the manufacturer's recommendations, using the “Mouse Signal Transduction PathwayFinder (89 genes including 5 housekeeping genes)” array in conjunction with the RT^2^ Profiler PCR Array System from SuperArray Bioscience (Frederick, MD). The results are presented as transcript levels relative to the levels in untreated control cells with average mRNA levels of five internal control genes, including beta-actin, used as the normalization control. To verify changes in gene expression, real-time PCR was carried out on 12 selected genes. All primers were designed based on nucleotide sequences retrieved from Genbank using the Primer Express software (PE Applied Biosystems) (Supplementary Table S1).

### Analyses

Genes that showed differences in their expression levels were, of at least 2.0 fold, selected for the different analyses (hierarchical cluster analysis, functional cluster analysis, biological pathway analysis). Hierarchical clustering (GENE CLUSTER v3.0) and display programs (TREE VIEW) were also used for analysis (http://rana.stanford.edu/software). We performed unsupervised hierarchical clustering based on the most variably expressed genes using the Euclidean distance as the similarity metric and the average linkage method as the between-cluster distance metric. A t-test was also performed to find genes that have changed between PDT alone and As_4_O_6_ alone, and PDT plus As_4_O_6_ treatment. Supervised clustering of experimental samples was performed by reducing the number of genes by statistical analysis. To classify the gene expression profiles, functional analyses and KEGG (Kyoto Encyclopedia for Genes and Genomes) pathway analyses (http://www.genome.jp/kegg/pathway.html) were carried out as previously described [35], [36]. To perform a KEGG analysis, differentially expressed genes of each treatment group were used for the calculation of their attribution to pre-defined KEGG signaling pathways and analyzed by pair-wise comparisons. The different number of genes were seen in a given pathway. The Ingenuity Pathway Analysis software (IPA, Ingenuity Systems, Mountain View, CA) was utilized to identify networks of interacting genes and other functional groups. Semantically consistent pathway relationships were modeled based on a continual, formal extraction from the public domain literature (www.ingenuity.com/products/pathways_knowledge.html).

### Evaluation of in vivo antitumor effect

TC-1 cells (2×10^5^) were injected subcutaneously into the right flank of C57BL/6 mice. Tumor-bearing mice were injected ip with 7.5 mg/kg of As_4_O_6_ and/or intravenously injected with Radachlorin (10 mg/kg). Three hours after injection, the animals were anaesthetized and the tumors were irradiated with 300 J/cm^2^ via a light fiber inserted into the tumor mass. Each group included 7 animals. Tumor volume was determined *in vivo* by external caliper using the pi-based ellipsoid volume formula (length x depth x width x 0.5233) [37], [38]. The average value and standard deviation are based on calculated tumor volumes from the eight mice in each group. To assess tumor response, tumor growth was recorded every 2–3 days for 25 days. Tumor growth was measured 2–3 times a week using calipers.

### Western blot analysis

TC-1 cells were treated with 3 uM of As_4_O_6_ and/or 0.15 ug/ml of Radachlorin. The cell lysates (approximately 30 μg of protein) were separated in 12% polyacrylamide SDS-gels and transferred to a nitrocellulose membrane (Schleicher & Schuell, Dassel, Germany). This was then immersed in blocking buffer (5% skim milk and 0.1% Tween 20 in PBS, pH 7.4) for 1 h at room temperature and incubated with primary antibodies: (SantaCruz Biotechnology, Inc., California, USA), p53 (1∶200), GaDD45 (1∶200), p21 (1∶200), and actin (1∶5000) in blocking buffer overnight at 4°C. After incubation, the membrane was probed with horseradish peroxidase-labeled anti-mouse IgG antibody (1∶5000) in PBS (containing of 0.05% Tween 20 and 5% skim milk powder) for 30 min at room temperature. The proteins in the membrane were detected by an enhanced chemiluminescence detection system (Amersham, Buckinghamshire, UK) and the bands were visualized by autoradiography using X-ray film (Amersham).

### Blood perfusion analysis after As_4_O_6_ treatment

Hoechst 33342 (Sigma-Aldrich) was dissolved in a sterile phosphate-buffered saline (PBS) at a concentration of 3 mg/ml and injected intravenously (i.v.) 0.10 ml/20 g mouse. TC-1 tumor-bearing mice (n = 3) were divided into 1, 3, 6, 12, 24, 48, 72, 168 h post-As_4_O_6_ treatment groups. As the control, three mice (i.p. injected PBS) used in each time point. One minute before killing, mice were perfused with 15 mg/kg Hoechst 33342. Tumors were excised and immediately frozen in liquid nitrogen. Frozen tissues were embedded into OCT cryofixative (Sakura Finetek, Janpan) and tumor cryo-sections were cut using a CM1850UV cryostat microtome (Leica, Germany) and air-dried. All the processes were performed quickly and samples were protected from direct expose to light. Hoechst 33342 distribution was assessed using a AX70, TR-62A02 fluorescent microscope (Olympus, Tokyo, Japan). A scan consisted of a field-by field movement of the scanning stage based on a selectable meander pattern, depending on the tumor size. In each field, the microscopic image was recorded and processed; during the image processing using the Image Pro Plus digital image analysis system (Media Cybernetics, USA). Each picture was converted into a gray scale and the positive range value was fixed, and the percentage of the positive area to the whole tumor area was determined.

### Immunohistochemistry

Immunohistochemistry was used to analyze the expression of CD31. Tumor tissues were fixed in 4% paraformaldehyde, embedded in paraffin, and sectioned at 4 μm thickness. Tumor sections were deparaffinized, rehydrated, and quenched with 3% hydrogen peroxide for 10 min at room temperature. The sections were incubated in protein blocking solution for 20 min before the addition of the primary antibody. The sections were incubated for 2 h at 37°C with a 1∶50 dilution of rabbit anti-mouse CD31 (Abcam, UK). After incubation, the sections were washed in PBS for 5 min and anti-rabbit secondary biotinylated antibody was applied. After washings, the avidin-biotin complex was then applied to the sections, followed by extensive washing steps. A diaminobenzidine chromogen kit (Abcam) was used to develop sections.

### Histopathology

Animals were treated with 10 mg/kg of As_4_O_6_. At selected time points, tumors were excised and fixed in 4% neutral formalin and embedded in paraffin. Paraffin-embedded tissues were sectioned for routine staining with hematoxylin and eosin.

### Real-time PCR microarray analysis with TC-1 tumor tissue

TC-1 cells (2×10^5^) were injected subcutaneously into the right flank of C57BL/6 mice. Tumor-bearing mice were injected ip with 7.5 mg/kg As_4_O_6_ and/or intravenously injected with Radachlorin (10 mg/kg). Three hours after injection, the animals were anaesthetized and the tumors were irradiated with 300 J/cm^2^ via a light fiber inserted into the tumor mass. One day after treatment, animals were sacrificed and tumor tissue was harvested. Total RNA was isolated from tumor tissue according to the manufacturer's recommendations, using TRIzol and the Absolutely RNA kit from Stratagene. Real-time PCR was carried out on 13 selected genes, as described previously.

### Treatments interaction analysis

To determine the nature of the interaction between Radachlorin/PDT and As_4_O_6_ treatment, the data from the MTT assay were analyzed as reported previously [34] using CalcuSyn V2.0 software, (Biosoft, Cambridge, UK) [39]. The interaction of treatments was quantified determining a combination index (CI). CI < or >1 indicated synergy or antagonism respectively, whereas a CI value of 1 indicates additivity [40].

### Statistical analysis

Statistical analysis included ANOVA and the Student's t-test. The values for the different groups were compared. *P* values of less than 0.05 were considered statistically significant.

## Supporting Information

Figure S1 In vitro cell growth inhibitory effects of As4O6 and/or Radachlorin/PDT on HaCaT, HeLa, and SiHa cells. Each cell was cultured with 3 uM of As4O6 and/or 0.15 ug/ml of Radachlorin/PDT, respectively. Cell viability was determined based on the MTT assay. Each bar represents a mean [± SD (vertical line)] of three replicates (n = 3).
(TIF)

Figure S2 Cell apoptosis analysis using Annexin V/PI staining. TC-1 cells were cultured with 3 uM of As4O6 and/or 0.15 ug/ml of Radachlorin/PDT for 1 day. Cell pellet was resuspended in 500 μl annexin V HEPES solution (10 mM HEPES-NaOH, pH 7.4, 140 mM NaCl, 2.5 mM CaCl2) and incubated on ice for 30 min in the dark. Cells were then washed once in ice-cold HEPES buffer and PI was added just before FACS analysis. The results were analyzed with a FACS.
(TIF)

Table S1 Primer sequences used for PCR assays.
(DOC)
