# Supplementary figures and images for: Synergistic Anti-Tumor Effects of Combination of Photodynamic Therapy and Arsenic Compound in Cervical Cancer Cells: In Vivo and In Vitro Studies
Source: PLoS One. 2012 Jun 8;7(6):e38583. doi: 10.1371/journal.pone.0038583 (PMC3371011; doi:10.1371/journal.pone.0038583)

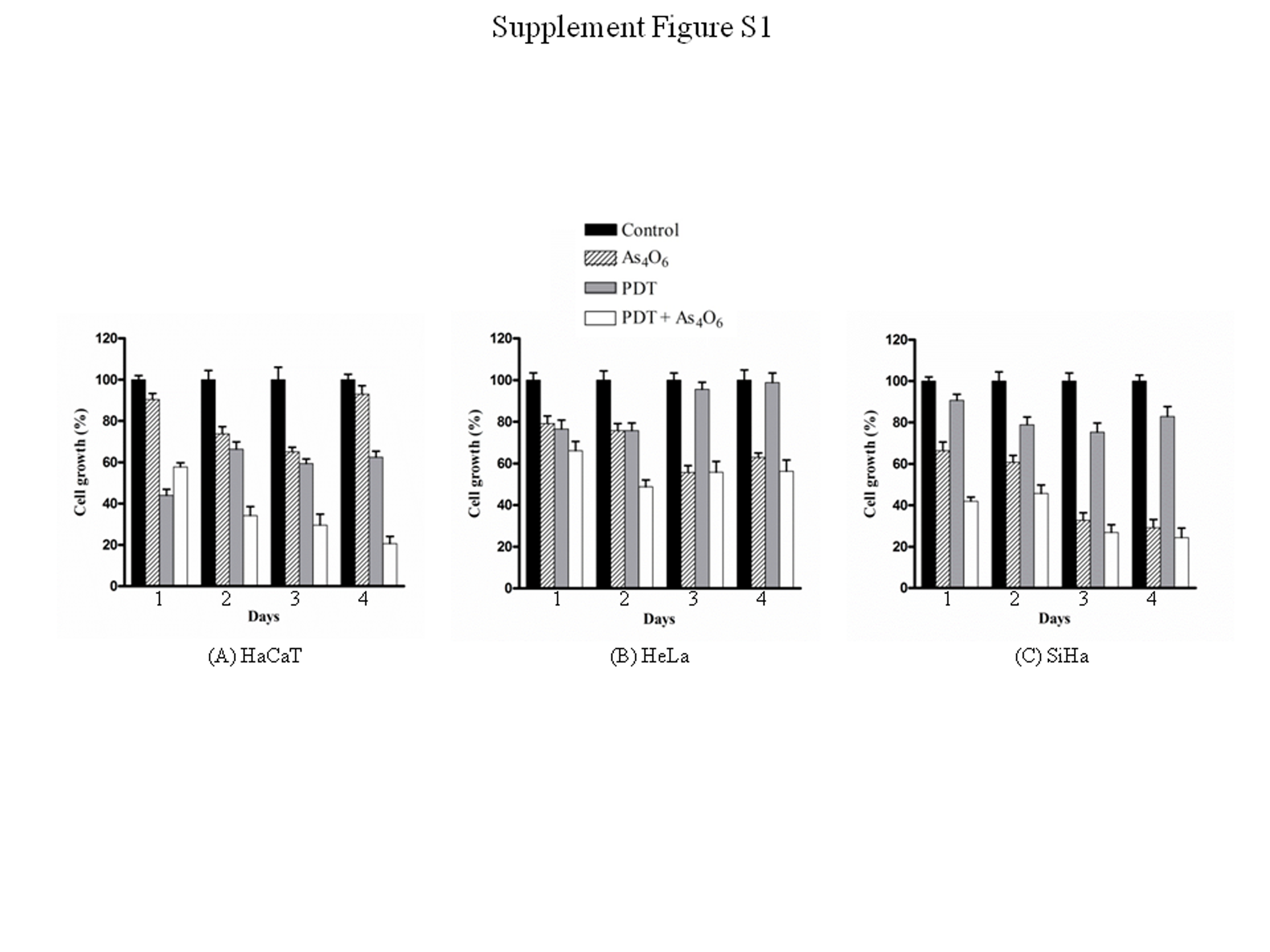

Supplement: Figure S1 — In vitro cell growth inhibitory effects of As4O6 and/or Radachlorin/PDT on HaCaT, HeLa, and SiHa cells. Each cell was cultured with 3 uM of As4O6 and/or 0.15 ug/ml of Radachlorin/PDT, respectively. Cell viability was determined based on the MTT assay. Each bar represents a mean [± SD (vertical line)] of three replicates (n = 3). (TIF) [file pone.0038583.s001.tif]

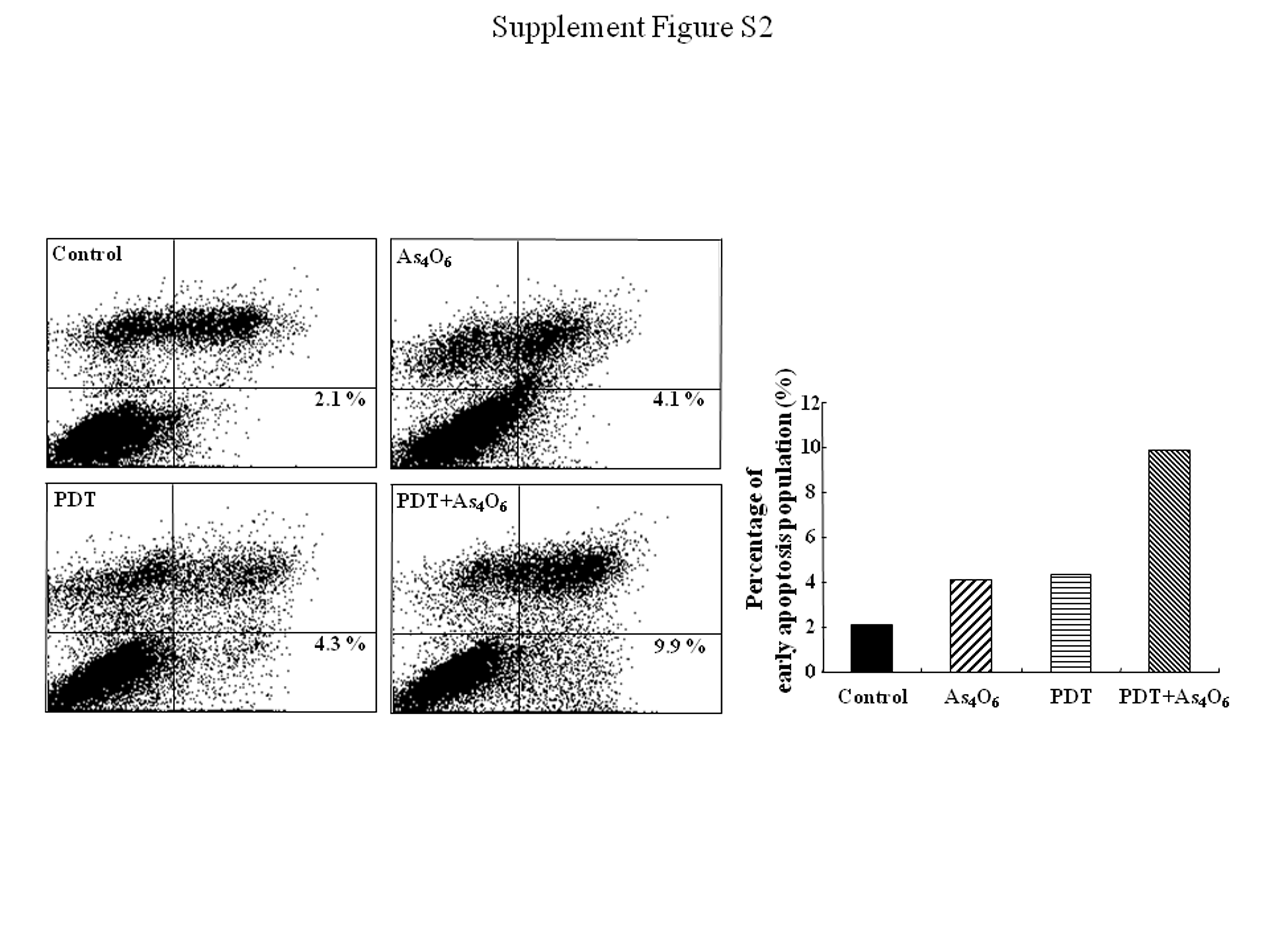

Supplement: Figure S2 — Cell apoptosis analysis using Annexin V/PI staining. TC-1 cells were cultured with 3 uM of As4O6 and/or 0.15 ug/ml of Radachlorin/PDT for 1 day. Cell pellet was resuspended in 500 μl annexin V HEPES solution (10 mM HEPES-NaOH, pH 7.4, 140 mM NaCl, 2.5 mM CaCl2) and incubated on ice for 30 min in the dark. Cells were then washed once in ice-cold HEPES buffer and PI was added just before FACS analysis. The results were analyzed with a FACS. (TIF) [file pone.0038583.s002.tif]
