# Supplementary material for: Synergistic Anti-Tumor Effects of Combination of Photodynamic Therapy and Arsenic Compound in Cervical Cancer Cells: In Vivo and In Vitro Studies
Source: PLoS One. 2012 Jun 8;7(6):e38583. doi: 10.1371/journal.pone.0038583 (PMC3371011; doi:10.1371/journal.pone.0038583)
Supplement: Table S1 — Primer sequences used for PCR assays. (DOC) [file pone.0038583.s003.doc]

Table S1. Primer sequences used for PCR assays

| Gene  Symbol | Sequence | Size (bp) |
| --- | --- | --- |
| ACTIN | forward 5'- GGC TGT ATT CCC CTC CAT CG | 154 |
|  | reverse 3'- CCA GTT GGT AAC AAT GCC ATG T |  |
| CD 5 | 5'-GGT GAT GCT AAG TGG CTC CAA | 203 |
|  | 3'-GGG GTC TGTTCA ATG AAG GGA A |  |
| P21 | 5'-CCT GGT GAT GTC CGA CCT G | 103 |
|  | 3'-CCA TGA GCG CAT CGC AAT C |  |
| MDM 2 | 5'-TGT CTA CCG AGG GTG | 110 |
|  | 3'-TCC AAC GGA CTT TAA CAA CTT CA |  |
| IL 2 | 5'-GTG CTC CTT GTC AAC AGC G | 171 |
|  | 3'-GGG GAG TTT CAG GTT CCT GTA |  |
| IGFBP 3 | 5'-CCA GGA AAC ATC AGT GAG TCC | 101 |
|  | 3'-GGA TGG AAC TTG GAA TCG GTC A |  |
| GADD 45 | 5'-CCG AAA GGA TGG ACA CGG TG | 121 |
|  | 3'-TTA TCG GGG TCT ACG TTG AGC |  |
| EI24 | 5'-AGG GGA ATC AAA GAC TCC ATC T | 111 |
|  | 3'-GGC CAA GAG GCT ACT TGC C |  |
| BIRC 3 | 5'-ACG CAG CAA TCG TGC ATT TTG | 181 |
|  | 3'-CCT ATA ACG AGG TCA CTG ACG G |  |
| BIRC 2 | 5'-TGT GGC CTG ATG TTG GAT AAC | 164 |
|  | 3'-GGT GAC GAA TGT GCA AAT CTA CT |  |
| TRP53 | 5'-GCG TAA ACG CTT CGA GAT GTT | 144 |
|  | 3'-TTT TTA TGG CGG GAA GTA GAC TG |  |
| TERT | 5'-GCA CTT TGG TTG CCC AAT G | 147 |
|  | 3'-GCA CGT TTC TCT CGT TGC G |  |
| NAIP | 5'-TGC CCA GTA TAT CCA CGG CTA T | 116 |
|  | 3'-AGA CGC TGT CGT TGC AGT AAG |  |
